# Neurophysiological evidence for whole form retrieval of complex derived words: a mismatch negativity study

**DOI:** 10.3389/fnhum.2014.00886

**Published:** 2014-11-06

**Authors:** Jeff Hanna, Friedemann Pulvermüller

**Affiliations:** Brain Language Laboratory, Department of Philosophy and Humanities, Freie Universität BerlinBerlin, Germany

**Keywords:** morphology, derivation, MMN, ERP, EEG, German

## Abstract

Complex words can be seen as combinations of elementary units, decomposable into stems and affixes according to morphological rules. Alternatively, complex forms may be stored as single lexical entries and accessed as whole forms. This study uses an event-related potential brain response capable of indexing both whole-form retrieval and combinatorial processing, the Mismatch Negativity (MMN), to investigate early brain activity elicited by morphologically complex derived words in German. We presented complex words consisting of stems “sicher” (secure), or “sauber” (clean) combined with abstract nominalizing derivational affixes -heit or -keit, to form either congruent derived words: “Sicherheit” (security) and “Sauberkeit” (cleanliness), or incongruent derived pseudowords: *“Sicherkeit”, and *“Sauberheit”. Using this orthogonal design, it was possible to record brain responses for -heit and -keit in both congruent and incongruent contexts, therefore balancing acoustic variance. Previous research has shown that incongruent combinations of symbols elicit a stronger MMN than congruent combinations, but that single words or constructions stored as whole forms elicit a stronger MMN than pseudowords or non-existent constructions. We found that congruent derived words elicited a stronger MMN than incongruent derived words, beginning about 150 ms after perception of the critical morpheme. This pattern of results is consistent with whole-form storage of morphologically complex derived words as lexical units, or mini-constructions. Using distributed source localization methods, the MMN enhancement for well-formed derivationally complex words appeared to be most prominent in the left inferior anterior-temporal, bilateral superior parietal and bilateral post-central, supra-marginal areas. In addition, neurophysiological results reflected the frequency of derived forms, thus providing further converging evidence for whole form storage and against a combinatorial mechanism.

## Introduction

Arguably, the defining characteristic of human language is the ability to iteratively combine units of meaning into more and more complex meaningful structures. The atomic meaning carriers are called *morphemes* and their combinations can be described by morphosyntactic rules. However, recent research in cognitive linguistics has cast doubt on the view that morphologically complex words are in all cases combined and assembled from their composite parts. Compelling arguments have been raised that at least a subset of the frequently used complex forms are stored as whole forms or mini-constructions in a lexicon or “constructicon” (Langacker, 1987; Goldberg, 2003). Consequently, these stored forms would be activated as whole units in the word recognition and language comprehension process. Such whole-form constructions may exist at the level of sentences (idioms, for example), phrases, or single, morphologically complex words.

In the present study we explore the processing of morphologically complex words bearing a derivational affix (e.g., calm-ness). As German is well-known for its rich derivational-morphological system, German derived word stimuli are well-suited for such investigations. Derivational affixes modify the meaning of a word and, in many cases change its lexical category. For example, English derivational affixes -ness and -dom are taken on by adjectives, and convert them into nouns (calm*ness*, free*dom*). The German affixes we used in this study, -heit and -keit, share this property of converting adjectives into nouns. Additional advantages of the German forms are their phonological similarity to each other and their often unpredictable pairing with word stems; nearly all adjectives only allow pairing with one of them and, in exemplary cases, no phonological criteria are available that could firmly determine the to-be-chosen affix (Fleischer and Barz, 2012). As it is not straightforward to formulate a unique set of algorithmic rules describing relationships between their stems and affixes that encompasses all cases, these linguistic forms appear as good candidates for exploring the possibility that complex words may be stored as whole forms.

Current theories of derivational processing range from total *obligatory decomposition*, where all derived forms are combined from their morphemes (Taft, 2004), to *dual-route* models allowing for both whole-form storage and composition, depending on linguistic properties of the word or the individuals’ cognitive systems, which vary, for example, in maturation or language exposure (Caramazza et al., 1985; Schreuder and Baayen, 1995; Clahsen, 1999; Pinker, 1999; Ullman, 2001). A large body of evidence in the domain of visual masked priming (Rastle and Davis, 2008, for review) indicates that derived words undergo an obligatory morphological decomposition at an early stage of processing, and not only in the expected case of semantically transparent, morphologically complex words (e.g., hunter = hunt + er), but also in semantically opaque cases, where the word has the appearance of a derived form, but is actually morphologically simplex (e.g., corner ~ = corn + er) (Longtin et al., 2003; Rastle et al., 2004). Results from masked visual priming fMRI studies showing modulation of brain activity by morphological relatedness in left inferior frontal gyrus (LIFG) (Bozic et al., 2007; Levy et al., 2008) or occipital areas (Gold and Rastle, 2007), have been interpreted in favor of this account, although such activation *per se* cannot speak to the issue of whether whole-form storage or rather combinatorial processes are brought about by derived forms. Results from priming tasks where primes are fully perceivable have been used to suggest that semantically transparent derived forms are typically decomposed into their morphological constituents (e.g., Marslen-Wilson and Warren, 1994; Rastle and Davis, 2008 for full review).

Much prior neurophysiological work on derived forms also supports the obligatory decomposition hypothesis, though in many cases with evidence for an active, whole-form access route being available under special circumstances, for example with semantically opaque items. Two studies found enhanced N400 components to incorrectly derived words (Janssen et al., 2006; Leminen et al., 2010), which can be seen as supporting decomposition, as the N400 is known to be enhanced to semantically incongruous combinations of words (Kutas and Federmeier, 2011). Another EEG study found a reduced N400-like component in response to morphologically complex target stimuli primed by forms sharing their stem with the targets, in comparison to prime-target pairs with no morphological relationship (Lavric et al., 2007), which was used to argue that the same morphological unit is included in both prime and target, thus supporting composition and combination. However, later studies showed that such relatively late effects, following the critical stimulus word by 400 ms and longer, are only present in specific tasks and that early brain responses are increased to congruent derived forms compared with forms that violate morphological regularities, thus going against the N400 pattern (Leminen et al., 2011, 2013a). Bölte et al. (2009) found that incongruently derived words produced a left anterior negativity (LAN), which is generally thought to reflect “syntactic” or combinatorial processing (Kutas et al., 2006). Other studies found an ERP/F component around 200 ms after stimulus presentation, which was modulated according to whether there was a potential morphological relationship between prime and target, but not whether this relationship was semantically transparent or opaque (Zweig and Pylkkänen, 2009; Lehtonen et al., 2011; Lavric et al., 2012), which was also used to argue in favor of obligatory decomposition of derived forms. Solomyak and Marantz (2010) found that M170 amplitude correlates with the transition probability of lemma to suffix, but found no correlation to bigram-based transition probabilities on the same items. The authors interpret this as consistent with obligatory decomposition. However, a follow-up study with more items and participants also found an accompanying effect for surface form frequency, suggesting a parallel, whole-form access route (Lewis et al., 2011). Finally, Leminen et al. (2011) compared inflectional and derivational morphology processing and found that while the former produced a tight, consistent left lateralized activation of cortical sources in the perisylvian language cortex, derived and simplex words sparked a more dispersed and bilateral network of sources, with stronger RH activity for derived than simplex and inflected words. The authors interpret this topographical difference as evidence for whole-form access of derived words, with the possibility that derived forms are also in some cases decomposed in parallel.

In sum, consistent with a major part of the linguistic literature, most of the past behavioral, neurophysiological and brain imaging research, largely done in the visual modality, seems to support obligatory decomposition of morphologically complex derived words. The handful of studies which used the spoken modality produced results more consistent with a dual route account, suggesting that at least under specific circumstances and early after the onset of the critical morphologically derived stimulus (100–300 ms), whole form access may become relevant (Leminen et al., 2010, 2011; Whiting et al., 2013). As a fundamental theoretical caveat, the rationale underlying the interpretation of brain activation results rely on heuristics which were not always straightforward. For example, an N400 increase was sometimes used as an argument for combinatorial (de)composition, although it is well-known that this brain response also distinguishes whole-form-stored words from novel and therefore not stored pseudo-words, so that it is not a unique indicator of either storage or combination (Kutas and Federmeier, 2011). Other questionable heuristics concern the brain loci activated: left inferior frontal activity was sometimes used as an argument for decomposition, although single word and construction processing engage this locus too (Pulvermüller et al., 2009; Allen et al., 2012; Bozic et al., 2013a). For these reasons, it is desirable to investigate the brain basis of derivationally complex words (i) using spoken language as the primary and native modality of language; and (ii) using a theoretically founded neuromechanistic rational for interpreting brain responses to language.

**Figure 1:**
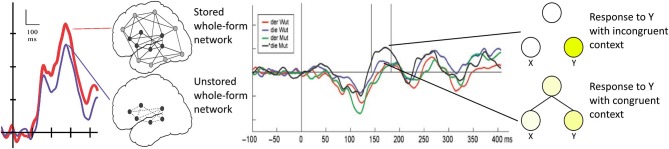
**Display of mechanisms and neurophysiological indices of whole form retrieval (left side) and combinatorial processing (right)**. Left: The neurobiological substrate of a whole-form-stored word or construction is seen as a strongly connected, distributed neuronal circuit, encompassing not only the word’s phonemic and acoustic properties (black nodes), but also its lexical and semantic properties (gray nodes). In constrast, an unfamiliar pseudo-word would activate only phonemic and acoustic networks, with comparatively weaker connections (dashed lines). Due to the broader connections of the construction circuit, it generates stronger activation than the weakly connected neuron set, as reflected in the differing strengths of their corresponding MMN brain responses. Right: The neurobiological basis of a regular combination of symbols is seen as a set of stand-alone circuits with strong combinatorial links between them. These strong between-circuit links are missing in case of a sequence that violates combinatorial regularities. When activated, the terminal member of the combinatorial circuit creates less activation than the terminal member in the incoherent circuit, because the priming between strongly-connected network members reduces the final activation enhancement needed to fully ignite the terminal element. This extra activation is reflected in higher MMNs for the terminal element of an ungrammatical string (MMN data adopted from Pulvermüller et al., 2001; Pulvermüller and Assadollahi, 2007).

Whole forms are stored by memory traces, which, at the neurobiological level, are neuronal circuits that develop when words and constructions are being learned (Pulvermüller and Fadiga, 2010). Neurocomputational simulations and neuroimaging work show that these neuronal circuits are typically distributed over several areas (Garagnani et al., 2008). Activation generated by these memory circuits may add to the activation provided by sensory stimulation, so that when familiar words are recognized, stronger overall brain activity is elicited compared with the processing of acoustically similar pseudo-words, which would not activate a corresponding distributed neuronal assembly (Pulvermüller et al., 2014). The neurophysiological difference between existing, stored forms and unstored, novel forms should therefore be relatively greater activation to the stored forms (Figure 1, left). In contrast, combinatorial processes are supported by mechanisms that apply the same algorithm or combinatorial schema to a whole class of stored item. At the neuromechanistic level, this mechanism is captured by combinatorial neuronal circuits linked with two or more sets of neuronal assemblies for stored items (Pulvermüller, 2010). In this case, the typical combinatorial context of a target word leads to pre-activation or priming of a target word’s representation, so that, when the word itself appears, its neuronal assembly is already active to a degree and the additional activation process to bring it to full ignition is therefore reduced compared with the unprimed case (Figure 1, right). The neurophysiological difference between forms that are connected by a combinatorial mechanism and unlinked ones is therefore relatively reduced activation for the former. Thus, whereas stored forms should *increase* the brain response relative to unstored ones, regularly-combined forms should elicit *smaller* brain responses than ill-combined ones. These reverse neurophysiological indicators of combination and storage are underpinned by explicit neurocomputational simulations and experimental results. In this context, one brain response has been particularly fruitful, the mismatch negativity, or MMN, as we will explain below.

The MMN is an ERP which indexes the perception of change, for example when a series of frequently presented identical “standard” stimuli is interrupted by a rarely appearing and therefore unexpected “deviant” stimulus (Näätänen et al., 2007). In comparison to the ERP responses to standard stimuli, the ERP response to deviant stimuli shows a negative deflection manifesting in the fronto-central electrodes, typically somewhere between 100–200 ms after acoustic deviance. Interestingly, it could be shown that this MMN response to spoken words and constructions shows exactly the dynamics to stored and combined forms predicted by the neuromechanistic model summarized above: words elicit larger MMN responses than acoustically and psycholinguistically matched, novel, pseudo-word syllable combinations. We call this extra MMN activation for words or whole-forms the “lexical MMN” (lMMN; Korpilahti et al., 2001; Pulvermüller et al., 2001, 2004; Kujala et al., 2002; Shtyrov and Pulvermüller, 2002; Endrass et al., 2004; Pettigrew et al., 2004; Shtyrov et al., 2005, 2010). On the other hand, grammatically congruent combinations of words and morphemes elicit *reduced* MMNs relative to the large ones elicited by ungrammatical strings, called here “syntactic MMNs” (sMMN), indexing lack of a combinatorial mechanism (Pulvermüller and Shtyrov, 2003; Shtyrov et al., 2003; Pulvermüller and Assadollahi, 2007; Herrmann et al., 2009; Bakker et al., 2013; Hanna et al., 2014). Therefore, the MMN offers the opportunity to address questions about storage and combination at the neurophysiological level.

Over and above its properties as a neurophysiological index of whole-form-storage and combination, the MMN brings several further advantages for neuroscience investigations into language. First it manifests early, within 100–200 ms after the critical information about a construction can first be distinguished and understood. This is important, because language comprehension is a fast and early process, and responses with longer latency therefore run into the problem that it can become difficult to decide whether any brain processes indexed are indeed a hallmark of first-access parsing and understanding, or are rather epiphenomenal (Pulvermüller et al., 2009). Second, the MMN is elicited regardless of whether participants focus their attention on the stimuli or elsewhere. This is important because natural language is mostly understood without effort; in fact, it is very difficult *not* to understand one’s native language. An ERP which disappears with the participant’s attention is therefore not likely to index natural language processing *per se*, but possibly metalinguistic, post-linguistic, or task-related processes. Additional strengths of linguistic MMN experiments are that they use orthogonal designs and make it possible to minimize the variance caused by acoustic variation. These features make the MMN an ideal tool for investigating higher linguistic and cognitive processes, and especially for looking at the brain basis of storage and combination (Pulvermüller and Shtyrov, 2006).

Because of its double potential as an index of both whole form storage and combination, the MMN has indeed recently been used to inform the linguistic debate around whole form retrieval vs. combinatorial processing of complex words and constructions. Looking at inflected forms, Bakker et al. (2013) found larger MMN responses for incongruently inflected past-tense forms, i.e., a sMMN, suggesting combinatorial processing for regular past-tense, rather than whole-form storage. Cappelle et al. (2010) found that particle verbs, in spite of their manifestation as different words dispersed over a sentence, still behave neurophysiologically as single, stored lexical items, with congruent particle verbs like “heat…up” producing stronger MMNs than incongruent ones like *“cool…up”. Leminen et al. (2013b), used an orthogonal MMN design to directly compare inflectional and derivational processing in Finnish and found not only the whole-form-storage index (lMMN) for derived forms, but also the combinatorial pattern (sMMN) for inflected forms. This would indicate a status as whole-form items for complex derived words, which goes against the body of evidence favoring (de-) composition and combination. As highlighted in the discussion above, data and opinions diverge about the status of semantically opaque complex forms, but it is relatively uncontroversial that semantically transparent complex derivational forms are seen as combined from their parts (Marslen-Wilson et al., 1994). To clarify the issue, we looked here at transparent derived forms in a language with rich derivational morphology, German.

The current study exploits the sMMN/lMMN to explore how German derived nouns are processed by native speakers. In German, an adjective may be rendered into an abstract noun by use of the derivational suffixes -heit and -keit (similar to English -ity or -ness). For example, “sicher” means “secure”, “sicherheit” means “security”, “sauber” means “clean”, “sauberkeit” means “cleanliness.” Note that these forms are semantically transparent so that classic morphological theories predict decomposition and combination. We presented “sicherheit”, *“sicherkeit”, *“sauberkeit”, and “sauberheit” as deviant stimuli in the context of standard stimuli “sicher” and “sauber”. When for example “sicher” is a standard, and “sicherheit” follows as a deviant, an MMN is elicited from the onset of the “h” sound, and will additionally be modulated either by its status as a real word, or its status as a morphosyntactically correct combination. When *“sicherkeit” follows as a deviant however, the MMN response will be modulated by the word’s status either as a pseudoword or an incongruent combination of morphemes. Any difference between these MMN responses however could easily be explained by the acoustic differences between “heit” and “keit”, so a further control condition is necessary. We accomplish this by introducing an experimental block where “sauber” replaces “sicher” as the standard stimulus and root lexeme in the deviant stimuli. In this case, -heit completes a pseudoword/incongruent combination and -keit completes a real word/congruent combination, thus yielding an orthogonal design in which additive effects of any of the stems or affixes cannot act as confounds. If the congruent forms “sicherheit” and “sauberkeit” produce stronger responses than the discordant ones, “*sicherkeit” and “*sauberheit”, the neurophysiological evidence speaks in favor of whole form retrieval. If the incongruent forms produce stronger responses however, there is a brain-based argument for combinatorial processing and decomposition.

## Materials and methods

### Design

**Figure 2:**
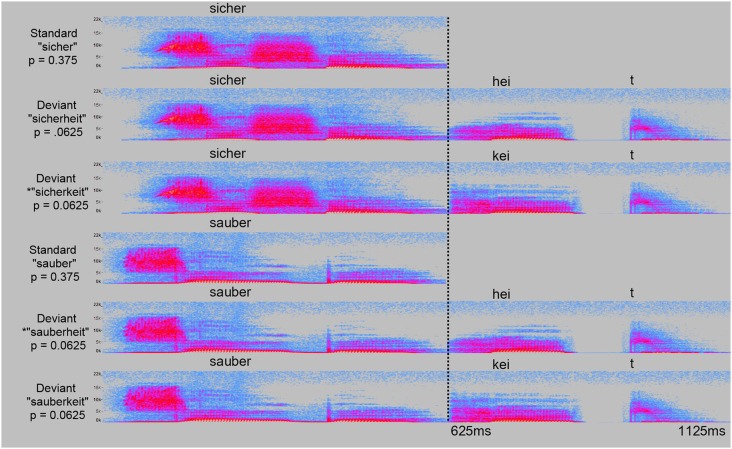
**Acoustic spectrograms of all stimuli**. For each experimental part, the standard stimulus (“sicher”, “sauber”) is shown together with the two deviant stimuli (“-heit”, “-keit”). Probability of occurrence in the experiment is indicated for each stimulus as probability “*p*”.

This experiment elicited MMNs using the classic, oddball paradigm where deviants occur rarely in a stream of more frequent standard stimuli. In this case, 1260 standards (3/4 of total stimuli), and 420 deviants. The stem (“sicher” or “sauber”) served as the standard in a given block, and the corresponding deviants were the stem appended with “-heit” or “-keit” (see Materials). The result is four deviants: sicherheit, *sicherkeit, *sauberheit, and sauberkeit (see Figure 2).

There were between three and five occurrences of standard stimuli between deviants, and an initial habituation period at the beginning of each block, where the standard was repeated 15 times consecutively. Brain responses to these 15 repetitions were not included in the ERP averages, nor were brain responses to the standard stimuli occurring immediately after a deviant stimulus.

“Sicher” and “Sauber” stimuli were segregated into separate blocks; each block contained 630 presentations of the standard stimuli, and two deviants presented 105 times each. There was a stimulus onset asynchrony (SOA) of 2 s. Block priority was counterbalanced across participants. Stimuli were presented with E-Prime 2.0^1^.

### Participants

We collected data from 33 participants, recruited from the student population of the Freie Universität Berlin, who were right-handed, as confirmed using the Edinburgh Handedness Inventory (Oldfield, 1971), and native speakers of German, and had no linguistic or neurological disorders. The experiments were performed with the approval of the Ethics committee of the Charité Universitätsmedizin, Campus Benjamin Franklin, Berlin.

### Stimuli

The MMN is highly sensitive to acoustic variation, so stimuli must be temporally aligned and identical, except where demanded by the parameters of the experiment. Toward this end, we recorded a female native speaker of German pronouncing “sicherheit” and “sauberkeit” several times—with a pause between the root and suffix to minimize coarticulatory bias in the root to a particular suffix—as well as the same stems followed by the word, “zeit.” We selected the “sicher” and “sauber” recordings that were most similar to each other in terms of length and peak sound energy as measured by acoustic wave forms and spectrograms, and eliminated remaining differences along these dimensions by selectively cutting the length of silence at the beginning of the recordings, with the result that they terminate at the same time, and by normalizing their sound energy to −5 dB after splicing (see below). Care was taken that stimuli shared the same intonational contour, as judged by a panel of three native German speakers listening to the stimuli candidates. In the same fashion, the most similar recordings of “heit” and “keit” were selected out. The final [t] morpheme was stripped out of both recordings and replaced by the [t] morpheme from a recording of “Zeit”. These edited “heit” and “keit” recordings were then spliced onto the “sicher” and “sauber” recordings. In order to achieve a natural intonation, the “heit” and “keit” recordings were reduced in amplitude by 5 dB, transposed down half a step in pitch, and the initial phoneme ([h] or [k]) was faded in from 50%–100% of the original volume. These steps smoothed the transition from the root into the suffix, resulting in stimuli that sounded like naturally pronounced, multi-morphemic words. Both standards were 625 ms long, and all deviants were 1125 ms long, with the “heit” or “keit” morpheme beginning at 625 ms. Sound recording and editing was performed with Audacity 2.0.3^2^. Acoustic spectra of the stimuli are shown in Figure 2.

According to the dlexDB psycholinguistic database for the German language (Heister et al., 2011), “Sicherheit” and “Sauberkeit” have normalized frequencies (n/million) of 116.5 and 5.4, respectively, and lemma frequencies of 118.3 and 5.4. “sicher” and “sauber” have 117.6 and 15.9, respectively, and lemma frequencies of 173.1 and 29.5. So “sicher” and “Sicherheit” are considerably more frequent than “sauber” and “Sauberkeit”.

### Procedure

Participants were seated in a comfortable chair facing a monitor, through which they watched a silent distractor movie with no linguistic content. They were instructed that they should ignore the acoustic stimuli, and may simply relax and watch the film. Stimuli were presented binaurally through high-quality headphones. The experiment lasted approximately 1 hour.

### EEG recording

Electroencephalogram data were recorded with 128 active electrodes (actiCAP system, BrainProducts, Gilching, Germany), with a ground electrode at AFz, and a reference electrode on the nose tip. Scalp electrodes were arranged in a modified 10–5 system, with occipital electrodes OI1 h, OI2 h, I1, and I2 removed. The electrooculogram (EOG), was recorded through three electrodes, two above and below the left eye, and one lateral to the right eye. The two vertical EOG electrodes were off-line re-referenced against each other to form the vertical EOG signal (vEOG), and this signal was then referenced against the third electrode to form the horizontal EOG signal (hEOG). Data were band-pass filtered (0.1–250 Hz) and sampled at 1000 Hz. Recordings were taken in an electrically and acoustically shielded chamber.

### EEG pre-processing

The following stages of pre-processing were carried out in EEGLAB 11.5.4.b^3^. Data were downsampled to 200 Hz, and bandpass filtered at 0.3–30 Hz. We then carried out a manual inspection of the data to remove bad channels and non-systematic bursts of noise. Electrooculogram channels were re-referenced offline as described above. Independent component analysis was used to derive 64 components from the data. Components which correlated with either vEOG or hEOG with *r* < −0.3 or *r* > 0.3 were removed from the data, thus significantly reducing eye-related artefacts. Removed channels were then spherically interpolated back into the data. Triggers used in the averaging process were set to the point where deviant stimuli first diverge acoustically from the standard stimuli, and moved forward 25 ms to compensate for the delay between trigger and auditory stimulus onset immanent to the stimulus delivery system. The continuous recording was then epoched into trials of 850 ms, starting 50 ms before the trigger and ending 800 ms after it. This 50 ms period before the trigger served as the baseline.

From this point, data were pre-processed in SPM8^4^. Epochs with a maximum—minimum voltage difference >120 µv or a >25 µv jump across two consecutive data points were removed, and the remaining trials were averaged into ERPs for each condition and subject. The mean and range of the number of remaining trials after cleaning and rejection are displayed below in Table 1.

**Table 1:** **Mean and range of ERP trials remaining after pre-processing**.

	Sicher	Sauber	Sicherheit	Sicherkeit	Sauberheit	Sauberkeit
Mean trials	380	379	94	96	95	94
Range trials	303–412	309–420	73–104	79–105	77–105	74–103

Participants who produced ERP signals with low signal-to-noise ratios were excluded from the pool. These were identified by reversing the polarity of half the epochs for all deviant stimuli, and averaging them. On the standard ERP assumption that the signal remains constant across trials, the signal in the half of the trials with reversed polarity would cancel the signal in the other half. Therefore the average of flipped and non-flipped trials would be the noise component of the ERP (Schimmel, 1967; Campos, unpublished). The average root mean square (RMS) of this noise was calculated for the *a priori* defined time window of interest (100–200 ms after acoustic deviance), and divided into the RMS of the ERP signal for the same time period, producing a signal to noise ratio (SNR). Five participants either had an SNR less than one, or a signal less than 1 µv, and a further two had excessive muscle artefacts. These participants’ data were therefore excluded, leaving 26 (four male) participants.

### Sensor space statistics

Sensor space data were analyzed in two ways. The first was the standard approach, where condition values for each participant were computed for each of the four deviant conditions (sicherheit, *sicherkeit, *sauberheit, sauberkeit) by taking the mean amplitude across the time windows and electrode configurations where deviant response amplitude was strongest. These mean values were entered into a repeated measures analyses of variance (ANOVA), with ROOT (sicher and sauber) and SUFFIX (-heit and -keit) as two-level factors.

The second method is cluster-based permutation on ERP data in a 3d-volume format, where spatial configuration of the electrodes as a flat surface comprise two dimensions, and peri-stimulus time comprises the 3rd dimension (Maris and Oostenveld, 2007). The relevant statistical tests were then performed on each voxel. Voxels where *p*-values were below a given threshold were grouped into clusters, and the “weight” of the clusters was determined by adding the *F*-ratios of all voxels in a given cluster together. In order to determine what cluster weights are likely to reflect real differences, a permutation-based Monte Carlo simulation is run, where for each iteration of the simulation, conditions are randomly distributed through the model, in effect simulating a null hypothesis. For each iteration the clusters are weighed, and the heaviest cluster is selected out. The distribution of these null-hypothesis cluster weights across many iterations (in our case, 1000 iterations) provides a measure of likelihood that the clusters found in the original statistical test are false positives.

### Source localization

Electrodes were co-registered in the standard 10–5 spatial configuration onto the scalp of the EEG boundary element forward model, based on the canonical MRI template included in SPM8.

For source localization, conditions were averaged according to congruency (“Sicherheit” and “Sauberkeit” vs. *“Sicherkeit” and *“Sauberheit”). Distributed source localization was carried out with the multiple sparse priors (MSP) approach (Friston et al., 2008) in SPM8. Group inversion was performed, thereby constraining spatial source solutions uniformly across participants (Litvak and Friston, 2008). Voxel images were produced summarizing the source activity at time points of interest (Figure 5B), and smoothed with a kernel size of 12 mm. These images were then submitted to their respective multi-voxel paired sample *t*-test.

**Figure 3:**
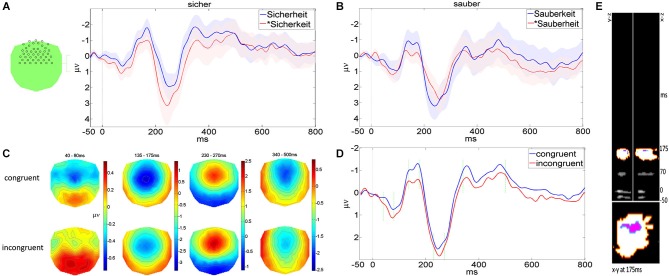
Signal space analysis (A) MMNs for Sicherheit, *Sicherkeit, shaded areas indicate 95% confidence intervals (B) MMNs for Sauberkeit, *Sauberheit and standard stimulus sauber, shaded areas indicate 95% confidence intervals. **(C)** Topographies of congruent (average of Sicherheit and Sauberkeit) and incongruent (average of *Sicherkeit and *Sauberheit) deviants, in the time windows selected for statistical comparison. **(D)** MMNs for congruent and incongruent conditions, green vertical lines indicate time windows for signal space analysis and topography display in **(C)**. **(E)** Results of voxel-wise factorial ANOVAs of ERP data, converted into a 3d-volume. *X* and *Y* axes represent 2d electrode positions, and *Z* axis represents time. Gray voxels are where interaction of ROOT and SUFFIX factors reached *p* < 0.05, the orange cluster survived multiple-comparisons correction, and the purple voxels are where planned comparisons showed stronger responses for congruent conditions in both “sicher” and “sauber” conditions.

**Figure 4:**
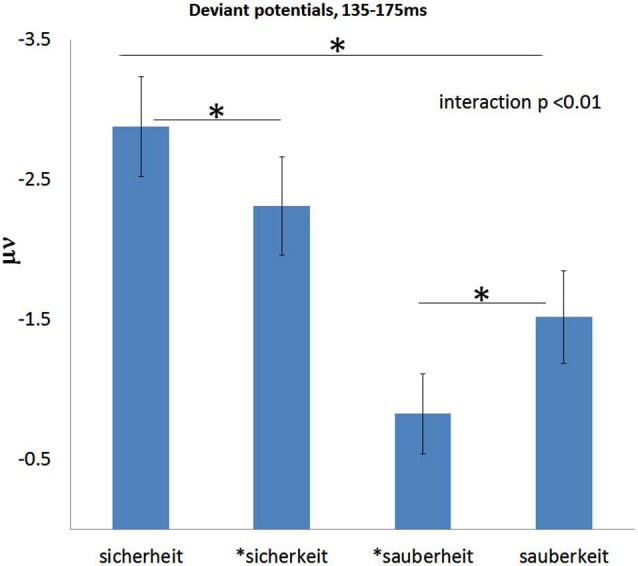
**Average deviant ERP voltages for the 135–175 ms time window measured at fronto-central channels obtained in the four conditions**. Note that for each pair and experimental block, the congruous condition elicits a significantly larger ERP than the infelicitous one. The interaction is signficant. Error bars are standard error of the mean.

**Figure 5:**
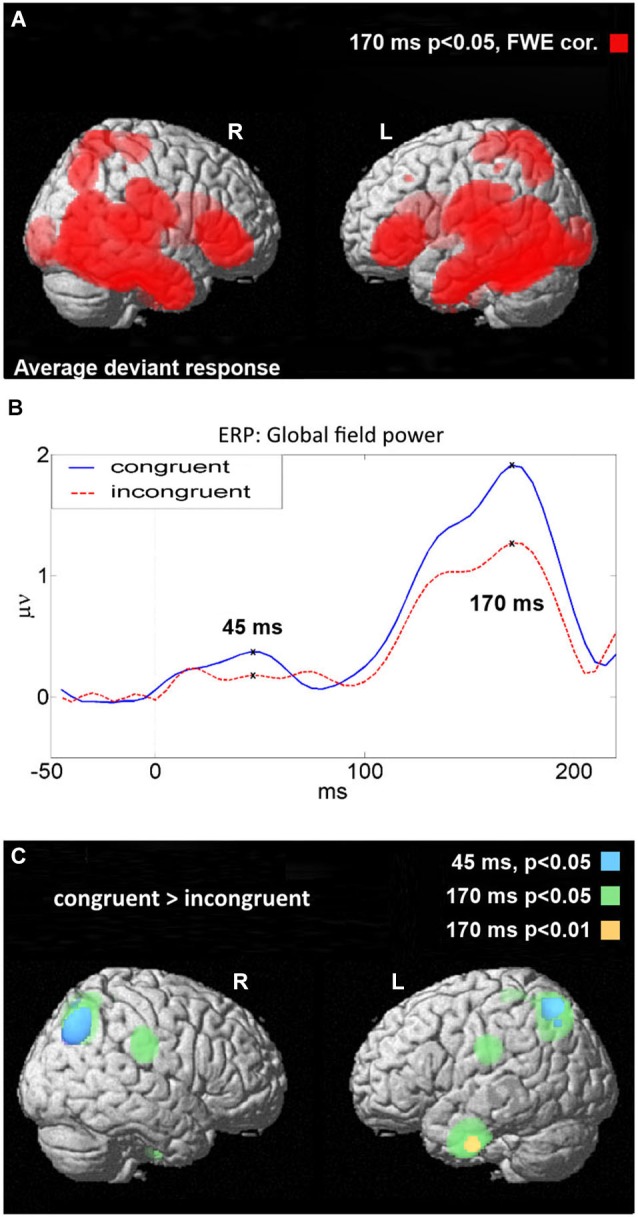
**(A)** Source activity for the average of all conditions, corrected at *p* < 0.05, adjusted for multiple comparisons. **(B)** Global ERP field power for congruent and incongruent conditions. The “X” markers indicate the time points of interest when source activity was analyzed. **(C)** Voxels where and when congruent conditions elicited a stronger response than incongruent conditions. All statistics uncorrected for multiple comparisons.

## Results

### Sensors

#### Standard analysis

Mismatch negativies (deviant minus its correponding standard) and deviant topographies for the four conditions are displayed in Figure 3 relative to a trigger point at the onset of the derivational suffixes “heit” or “keit”, where the acoustic waveforms of the standard and deviant stimuli first differed. Topographies show that the negative deflections occurred in fronto-central electrodes, as is typical for acoustic MMN paradigms, so waveforms used for display and statistics were calculated from the average of 46 electrodes in this area (pictured in Figure 3A). Note that MMNs are displayed in Figures 3A,B,D, but all analysis and source localization was carried out directly on the unsubtracted deviants.

The main MMN deflection emerged between 135–175 ms after acoustic divergence. In addition to this 40 ms-wide window, we investigated several other peaks for sensitivity to linguistic processes: a very early negative deflection (40–80 ms), a large positive deflection directly following the MMN (230–270 ms), and a late, extended negative deflection (340–500 ms).

At the main MMN peak (135–175 ms; Figure 4), congruent derived words produce stronger responses in both “sicher” and “sauber” conditions, and “sicher” conditions produce stronger responses than “sauber” conditions. Statistical results confirmed this impression. A 2 × 2 ANOVA with ROOT (sicher, sauber) and SUFFIX (-heit,-keit) as factors revealed a significant cross-over interaction of ROOT and SUFFIX (*F*_(1,25)_ = 9.5, *p* = 0.005). Planned comparisons showed that “sicherheit” produced a reliably stronger response than *“sicherkeit” (*F*_(1,25)_ = 4.4, *p* = 0.045), and “sauberkeit” produced a reliably stronger response than *“sauberheit” (*F*_(1,25)_ = 5.3, *p* = 0.03). In addition, the main effect of ROOT was significant (*F*_(1,25)_ = 5, *p* = 0.035), indicating that, on average, “sicher” conditions produced stronger responses than “sauber” conditions.

Already in the earlier time window (40–80 ms) there was a negative deflection, with the congruent deviants producing a seemingly stronger signal than the incongruent deviants. This pattern was marginally significant, with a cross-over interaction of *F*_(1,25)_ = 3.2, *p* = 0.086. There were no significant main effects.

In the later positive deflection (230–270 ms) there was a much stronger response to sicher and -keit conditions compared with their respective sister forms, confirmed by a main effect of ROOT (*F*_(1,25)_ = 13.4, *p* < 0.001) and SUFFIX (*F*_(1,25)_ = 6, *p* = 0.021). There was no interaction of these factors.

In the latest time window (340–500 ms), the negative deflection yielded only a main effect of ROOT (*F*_(1,25)_ = 21.92, *p* < 0.001).

#### Cluster-based permutation

Figure 3E shows the results of the same repeated measures described in the previous section, applied voxel-wise to ERP data in 3d-volume format. Gray-scale voxels show uncorrected *F*-values on the interaction of ROOT and SUFFIX, thresholded at *F* > 4.24 (*p* < 0.05, df = 1.25). When these voxels were grouped into clusters, one cluster (shown in orange on Figure 3E), corresponding to the 135–175 ms time window, was heavier than 965 of the 1000 maximum cluster weights in the Monte Carlo simulation of the null hypothesis (*p* = 0.035). No other cluster passed the *p* < 0.05 threshold. The area shown in purple on Figure 3E, corresponding to fronto-central electrodes at around 165–175 ms, is where planned comparisons showed that both the Sicherheit response was significantly more negative than the *Sicherkeit response, and the Sauberkeit response was significantly more negative than *Sauberheit (*p* < 0.05 in both cases).

### Sources

For all conditions generally, sources were concentrated in classical language areas: perisylvian, temporo-parietal, and inferior frontal gyrus, in both hemispheres, as thresholded at *p* < 0.05, family-wise corrected with random field theory (Brett et al., 2004; Figure 5A). For statistical comparisons between congruent and incongruent conditions, we focused on those time windows when ROOT and SUFFIX interacted, namely 40–80 ms and 135–175 ms, and produced voxel images summarizing source activity at each time window’s peak global field power, 45 ms and 170 ms, respectively (Figure 5B). Unidirectional, voxel-wise *t*-tests on these images found that at 170 ms, congruent deviants produced stronger responses than incongruent deviants at clusters in bilateral superior parietal regions, bilateral central/post-central/supramarginal regions, and left superior post-central regions (*p* < 0.05, uncorrected), as well as a difference in the left middle/inferior temporal gyrus (*p* < 0.01, uncorrected). At 45 ms, two clusters in bilateral superior parietal regions were significant (*p* < 0.05, uncorrected), contained entirely within the parietal clusters active at 170 ms. Cluster peak coordinates are summarized in Figure 5C and Table 2. Unidirectional *t-tests* in the other direction (incongruent stronger than congruent) produced no significant voxels at either time point. We stress here that statistical comparisons between congruent and incongruent conditions did not survive whole-brain family-wise error correction, and so should be intepreted with appropriate caution.

**Table 2:** **Source statistics summary for congruent > incongruent comparison, all statistics uncorrected for multiple comparisons**.

Cluster region	Time point (ms)	Statistics at peak	MNI
Left superior parietal	45	*T*_(1,25)_ = 1.79, *p* < 0.05	−24 −62 56
Right superior parietal	45	*T*_(1,25)_ = 1.82, *p* < 0.05	42 −72 36
Left inferior temporal	170	*T*_(1,25)_ = 2.54, *p* < 0.01	−46 −10 −36
Left superior parietal	170	*T*_(1,25)_ = 2.3, *p* < 0.05	−24 −64 54
Right superior parietal	170	*T*_(1,25)_ = 2.2, *p* < 0.05	34 −64 46
Left central sulcus/postcentral/supramarginal	170	*T*_(1,25)_ = 1.8, *p* < 0.05	−50 −22 28
Left superior postcentral	170	*T*_(1,25)_ = 1.76, *p* < 0.05	−19 −36 64
Right central sulcus/postcentral/supramarginal	170	*T*_(1,25)_ = 1.71, *p* < 0.05	56 −20 26

## Discussion

Derived words including stem and affix consistently produced stronger ERP responses than incongruent sequences of the same stems and derivational affixes, a pattern consistent with an lMMN, and therefore whole-form storage. Uncorrected source localization indicated that the generators underlying the enhancement of the MMN response to congruent relative to incongruent forms, the lMMN, were located primarily in bilateral posterior-parietal areas (angular gyrus), the left inferior temporal gyrus, and pericentral sensorimotor areas extending into anterior supramarginal gyrus. There was also a weaker, marginally significant “pre-lMMN” effect at 40–80 ms, localized in bilateral parietal areas.

### Derived forms are stored as whole-forms, not combined

Our present results show the brain activation correlates of whole-form storage for derived German words. Therefore, the data can be used to argue that the brain mechanisms sparked by these forms are those of stored whole form retrieval. In contrast, standard grammar theories and psycholinguistic models viewing derivation as a combinatorial process are not supported by these data (for discussion of psycholinguistic implications, see below). The present results cohere with prior studies that used the MMN to study derivational processing. Leminen et al. (2013b) also found larger MMNs to congruent derived forms of Finnish than to incongruent combinations, thus revealing the same neurophysiological signature of stored-form-retrieval as our present data on German nouns do. Leminen et al.’s derivational whole-form-storage MMNs were generated in left temporal areas, as ours here, and these authors also reported that their high-frequency derived words produced a larger MMN in comparison to low-frequency derived words. Whiting et al. (2013) localized MMNs for derived English words to the left middle temporal lobe, again where the lMMN enhancement was most reliably localized in our present study.

The cortical sources of MMN responses to stored linguistic forms and especially the activation enhancement for stored over unstored forms (“lexical MMN” or lMMN) have previously been localized in a range of different areas, most commonly in left or bilateral superior-temporal regions (Pulvermüller et al., 2001, 2004; Shtyrov et al., 2005). Inferior-frontal sources were seen especially for words and constructions semantically related to actions (e.g., Shtyrov et al., 2004; Pulvermüller et al., 2005; Pulvermüller and Shtyrov, 2009). Posterior-inferior parietal sources have been reported too, with special emphasis that these can vary between words (Pulvermüller et al., 2004); parietal sources were previously seen to be pronounced to prepositions and verb particles (Cappelle et al., 2010). This pre-existing research shows that localization of the lexical enhancement can vary substantially in its brain topography, and it appears plausible that this variability depends, in part, on lexical and psycholinguistic features of the particular word stimuli probed (Pulvermüller et al., 2009). Our present results show overall activation to linguistic stimuli across all the regions previously found active in this type of experiment (Figures 5A,C, *p* < 0.05, FWE-corrected), including superior-temporal, inferior-frontal and inferior-parietal areas within the perisylvian language cortex and also dorsolateral central cortex, posterior parietal cortex and inferior temporal lobe outside, in “extrasylvian” space. However, amongst these areas generally active to both congruent and incongruent forms, only a subset seemed more active to congruent than to incongruent forms ending in a derivation suffix. These were the extrasylvian parietal and temporal areas around the angular gyrus and the temporal pole, both known as areas that have recently been proposed as “semantic hubs” that process meaning-related information (Patterson et al., 2007; Pulvermüller, 2013). In addition, lMMN sources in perisylvian frontocentral sensorimotor cortex and anterior supramarginal gyrus may suggest action-related meaning processes. Still, we have to warn against giving these results any strong interpretation, as the levels of significance at which between-condition differences in source space could be documented were low (*p* < 0.01 or 0.05), and still more importantly, did not pass family-wise error correction—in spite of the clear and significant differences in signal space. Regardless of the precise interpretation of the source dynamics, the results seem to speak against the involvement of combinatorial processes. The sMMN to ungrammatical strings, which we take as evidence for a combinatorial process, has its typical sources in left superior temporal areas with MEG (Shtyrov et al., 2003; Pulvermüller and Assadollahi, 2007; Herrmann et al., 2009; Bakker et al., 2013) and left inferior frontal areas with EEG (Pulvermüller and Shtyrov, 2003; Hanna et al., 2014), but not where the current source analysis suggested generator differences between conditions. As inferior-temporal and posterior-parietal sources are typical for semantic brain activity frequently seen to single words, whereas combinatorial-linguistic processes usually have a perisylvian signature, the present source pattern supports the lexical whole-form storage interpretation.

Given the tentative nature of our present localizations of lMMN sources obtained for derived words, and of any neurophysiological source localization generally (Hämäläinen et al., 1994), it is important to note that the suggested activation loci agree with those of two recent fMRI studies which focused specifically on derivational morphology processing to auditory stimuli. These studies consistently found activation in bilateral middle temporal lobes (Bozic et al., 2013a,b) when brain responses to derived forms were compared with inflected forms. Our present results, demonstrating left anterior inferior and bilateral middle-temporal activation enhancements to congruent derived forms compared with incongruent forms, show a reasonable agreement with these authors’ main findings.

Even though the words used in the present study were not matched for all psycholinguistic factors that could potentially affect the brain response, one of them is clearly more common and more frequently used than the other in standard German (dlexDB normalized word frequencies 116.5 vs.5.4). It is therefore noteworthy that, consistent with previous results (Alexandrov et al., 2011; Shtyrov et al., 2011), a stronger MMN emerged for the more frequent item (“Sicherheit”). The frequency sensitivity of the MMN suggested by the present data provides a further indication that we measured a whole-form retrieval process and not a combinatorial one. Word frequency is one of the oldest and most robust test variables for lexical status, widely measured in behavioral tasks (e.g., Balota et al., 2004), metabolic neuroimaging (e.g., Hauk et al., 2008), ERPs (e.g., Hauk et al., 2006; Kutas and Federmeier, 2011; Shtyrov et al., 2011), and specifically is also indexed by MMN to monomorphemic words (Alexandrov et al., 2011; Leminen et al., 2013b). Brain responses indexing combinatorial processes invoked by inflectional and syntactic mechanisms by contrast do not seem to be affected by the frequency of their lexical roots (Pulvermüller and Assadollahi, 2007; Leminen et al., 2013b). The frequency-independence of combinatorial processes which can be described using algorithmic rules is a well-known phenomenon supported by substantial psycholinguistic evidence (Pinker, 1997). The neuromechanistic basis for the frequency-sensitivity of whole-form access can be theoretically grounded in the postulate that whole forms are stored as distributed neuronal assemblies that become more frequently connected internally the more frequently they are activated together, thus yielding more strongly connected assemblies for high-frequency words and constructions than for low-frequency ones (Pulvermüller, 1999). Activation dynamics reflect connection strength producing stronger activation with stronger links. In contrast, combinatorial processes rely on mechanisms binding information across large groups of lexical items so that the combinatorial links apply equally to high- and low-frequency items and are therefore independent of the frequency of a particular sequence of words (Pulvermüller, 2010).

### Implications for psycholinguistic theories

These results seem to argue against psycholinguistic models of obligatory decomposition (Clahsen et al., 2003; Marslen-Wilson, 2007). Even if such models allow for secondary whole-form access under special circumstances, i.e., a “rules and words” framework (Pinker, 1997), it would need to be explained why two morphologically different German words show the neurobiological signature of whole-form access and retrieval at earliest latencies (135–175 ms), with marginally significant foreshadowing of such difference already at *ca*. 40 ms, and why similar previous studies by Leminen et al. (2013b) revealed the comparable results for derived forms of Finnish. The special significance of this present study in German comes from the complexity of the morphological rules and construction schemes underlying the forms “Sicherheit” and “Sauberkeit”. According to standard German morphology and grammar (Fleischer and Barz, 2012), there is a semi-regularity according to which a bisyllabic adjective ending in the syllable “er” (common to both of stems here) tends toward the nominalizing derivational suffix -keit, not -heit. The assumption of such a regular pattern is supported by the fact that many more nominalizations of bisyllabic er-adjectives take -keit than -heit. “Sauberkeit” could therefore be an instance of a rule-combined form. For nouns including an “er” adjective with two syllables and -heit, the argument can therefore be made that they represent exceptions from the “keit-rule” and can therefore be regarded as whole-form-stored mini-constructions. Such exceptional whole-form storage should therefore apply to “Sicherheit.” The prediction of this theory is that the brain dynamics elicited by congruent -keit forms are those of combination and composition, whereas those to -heit forms should index whole form storage. In showing the whole form storage pattern is elicited by both types, our results speak against this “mixed” account.

However, it must be pointed out here that while “Sicher” and derivatives are much more frequent than “Sauber” and derivatives, both are quite frequent in German. It may be that when very infrequent words are tested against frequent words, a combinatorial mechanism is used in ther former, and a whole-form mechanism in the latter. This remains a promising avenue for future research.

At the level of linguistic theory, the present results seem to sit comfortably with current approaches to construction grammar according to which a large repertoire of constructions can be learned and stored from experience (Goldberg, 2003). In this approach, derived forms would be considered mini-constructions stored on an item-by-item basis, based on general neurobiological laws such as Hebbian learning (Pulvermüller, 1999). It is clear that, if linguistic forms are frequently recombined with each other, this combinatorial information is also mapped at the biological circuit level so that combination schemas are created. The neurobiological mechanisms for such formation of combinatorial schemas has been explored with neurocomputational network simulations and the linguistic theory for such schemas particularly well developed in the domain of argument structure constructions (Goldberg, 2006). This research encourages future empirical questions, especially ones about the cause behind the shift between storage of single whole forms and the development of a combinatorial schema and structural construction.

The most probable reason for the discrepancy between the dominating opinion in psycholinguistics and our present findings is that most studies that investigated this issue in the past used written stimuli, whereas we used auditory stimuli. While spoken and orthographic speech clearly must at some point share common linguistic substrates, they also must use distinct systems, and this is more likely to be so in the earlier stages of processing. Processing of written language also relies partly on visual object identification systems, further shaped by the non-innate capability to read and write (Rastle and Davis, 2008). We recommend then that this imbalance should be corrected, with further research on early-stage neurophysiology of morphological processing in the auditory modality.

### MMN as a tool for psycholinguistic investigation

Neuroscience research on the psycholinguistic question about whole-form retrieval or combinatorial processing of complex symbols and constructions requires a brain response that shows different dynamics to the fundamentally different types of predictions these mechanisms entail. Whole construction retrieval of a complex form AB implies that single representation or neuronal circuit is activated partially by utterance part A and the second utterance part B fully “ignites” the unitary AB circuit. The ignition of the larger circuit AB produces more activation than the activation of the composite circuit B on its own. In sharp contrast to this dynamic, a combinatorial mechanism connecting forms A and B implies separate autonomous mechanisms for the processing of both constituents and a functional combinatorial link between them. In this case, utterance part A activates its own circuit, which, in turn leads to partial activation (priming) of circuit B by way of the combinatorial mechanism. When B appears in this combinatorial congruent context, its circuit is already pre-active and therefore its full ignition leads to less activation relative to the pre-B baseline than when B appears in an incongruent context, where no combinatorial priming is present. As to the best of our knowledge, the only brain response that reflects this difference between storage-related and combinatorial mechanisms of prediction and processing in different and opposite dynamics is the MMN. Most other brain responses that have been successfully used to investigate language and cognitive processing show a “surprise signature” according to which the less expected event leads to increased amplitudes relative to the expected or predicted one. This expectancy violation or prediction error signature is well-documented for event-related responses including the N1 and P300 (sensory expectation and attention), N400 (lexical or semantic expectation), and ELAN, LAN and P600 (syntactic expectation) (Donchin, 1981; Neville et al., 1991; Osterhout et al., 1997; Kutas et al., 2006; Näätänen et al., 2007; Kutas and Federmeier, 2011). The opposite dynamics of the MMN to whole-form retrieval and combinatorial processing also makes it possible to obtain information from neuroimaging experiments about the cortical loci of activation, which may also provide clues about the storage-related or combinatorial nature of the neurocognitive processes. Looking back at the surprising set of results recently revealed by linguistic MMN research—including the evidence for combinatorial processing of inflected forms, whole form—retrieval of derived ones and whole-form storage of particle verbs (Cappelle et al., 2010; Bakker et al., 2013; Leminen et al., 2013b), this response offers itself as a fruitful tool for future investigation of the neurobiological basis of words, constructions and meaningful communication generally.

## Conclusion

We investigated early, automatic brain responses to derived words in German using the lMMN. The results indicate such words are processed as whole forms, evidenced as follows:

1. Congruent forms produce stronger MMNs than incongruent forms, consistent with MMN responses to stored whole-form items such as words or mini-constructions, which are enhanced relative to incongruous forms such as meaningless, unfamiliar pseudowords.
2. The MMN enhancement to the congruent forms relative to incongruous ones was localized to the left inferior-temporal and bilateral posterior-parietal and frontocentral sensorimotor areas. Thus in brain areas know for their role in word retrieval and semantic processing.
3. Frequent words produced stronger responses than infrequent ones, which is consistent with the well-known frequency sensitivity of word processing revealed by behavioral and neurophysiological studies.

In sum, these findings provide new evidence for a robust whole-form access route in the auditory perception of derived words—even highly transparent, productive ones—in the form of enhanced MMNs for existing, derived words, presumably reflecting extra activation from their lexical memory circuits. We hope these results shed new light on a crucial linguistic and psychological issue, namely the interplay between stored units or forms, and the combinatorial mechanisms which productively combine them.

## Conflict of interest statement

The authors declare that the research was conducted in the absence of any commercial or financial relationships that could be construed as a potential conflict of interest.

## Footnotes

^1^http://www.pstnet.com/eprime.cfm

^2^http://audacity.sourceforge.net/

^3^http://sccn.ucsd.edu/eeglab/

^4^http://www.fil.ion.ucl.ac.uk/spm/software/spm8/
